# Case Report: Missing zinc finger domains: hemophagocytic lymphohistiocytosis in a GATA2 deficiency patient triggered by non-tuberculous mycobacteriosis

**DOI:** 10.3389/fimmu.2023.1191757

**Published:** 2023-08-23

**Authors:** Xin Huang, Bingxuan Wu, Di Wu, Xiaoming Huang, Min Shen

**Affiliations:** ^1^Department of Rheumatology and Clinical Immunology, Chinese Academy of Medical Sciences & Peking Union Medical College, National Clinical Research Center for Dermatologic and Immunologic Diseases (NCRC-DID), Ministry of Science & Technology, State Key Laboratory of Complex Severe and Rare Diseases, Peking Union Medical College Hospital (PUMCH), Key Laboratory of Rheumatology and Clinical Immunology, Ministry of Education, Beijing, China; ^2^Department of General Internal Medicine, Peking Union Medical College Hospital, Chinese Academy of Medical Science & Peking Union Medical College, Beijing, China

**Keywords:** hemophagocytic lymphohistiocytosis (HLH), GATA2 deficiency, zinc finger domain, GATA2, Mycobacterium avium

## Abstract

Haploinsufficiency of GATA2, also known as GATA2 deficiency, leads to a wide spectrum of clinical manifestations. Here we described another 28-year-old man with a GATA2 variant who also suffered from hemophagocytic lymphohistiocytosis(HLH), who was finally diagnosed with HLH triggered by *Mycobacterium avium* bloodstream infection due to primary immunodeficiency. We reviewed GATA2 deficiency patients with HLH and found that GATA2 variants causing loss of zinc finger domains were associated with HLH, and erythema nodosa might be an accompanying symptom.

## Introduction

Hemophagocytic lymphohistiocytosis (HLH) is a life-threatening syndrome due to immune responses of activated macrophages and lymphocytes, with common clinical features including fever, splenomegaly, cytopenia, elevated aminotransferase and ferritin levels (1). Driven by the aberrant immune response, HLH can occur at all ages. The products of HLH-related genes are involved in cell-mediated cytotoxicity and lymphocytes activation/survival, and immunocompromised patients are susceptible to HLH (1). As an important determinant of multilineage hematopoiesis, GATA-binding protein 2 (GATA2) is a member of the GATA family of transcription factors.

Caused by heterozygous loss-of-function *GATA2* gene variants, haploinsufficiency of GATA2, also known as GATA2 deficiency, leads to a wide spectrum of clinical manifestations including mycobacterial infections, viral infections, bone marrow failure, leukemia and lymphedema (2). It has been reported that non-tuberculous mycobacterial infections were the most common infections, while disseminated mycobacteriosis was rare in childhood but becomes more frequent with age, due to the diminishing hematopoietic function of the bone marrow (3–5). GATA2 deficiency has highly variable penetrance, and infections or other factors may affect epigenetic mechanisms to trigger pathogenesis and alter penetrance (6).

In 2021, our group reported a 17-year-old Chinese Han woman with a heterozygous *GATA2* variant, who had recurrent HLH and erythema nodosa (7). Here we described another 28-year-old man with a *GATA2* variant who also suffered from HLH triggered by non-tuberculous mycobacterial infection, and further functional evaluation was conducted.

## Case description

A 28-year-old Chinese Han man was admitted with a history of intermittent fever for 3 years and erythema nodosa for 7 months. He had the first unprovoked onset of fever with a maximum temperature of 40°C lasting for two weeks. He had a second flare after two months and went to the local hospital, where he was diagnosed with hemophagocytosis and right inferior lobar pneumonia, based on bone marrow smear and chest computed tomography (CT). In 2021, he developed bilateral parotid enlargement, recurrent fever, and erythema nodosa. He was treated with various anti-infective agents, including moxifloxacin, linezolid, vancomycin, voriconazole, and cefoperazone-sulbactam, but the fever continued, mostly in the afternoon and at night.

**Figure 1:**
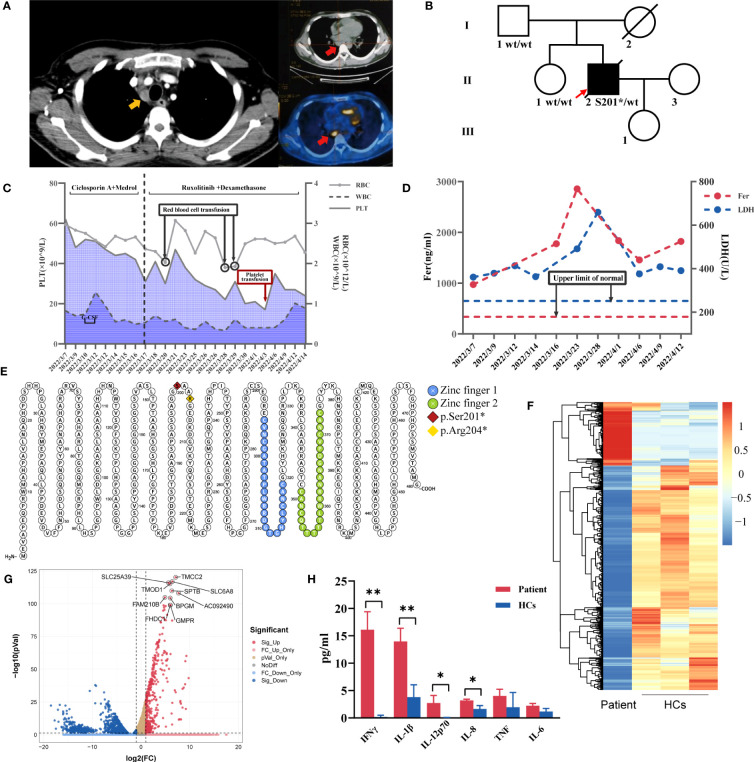
**(A)** Chest CT showing enlarged mediastinal lymph nodes (yellow arrow) and PET/CT with hypermetabolic mediastinal lymph nodes (red arrow). **(B)**Pedigree chart of the patient. **(C)** Trends in red blood cells (RBC), white blood cells (WBC), platelet count (PLT) levels and treatments during the patient’s hospital stay. **(D)** Changes in ferritin (Fer) and lactate dehydrogenase (LDH) during the hospitalization. **(E)** *GATA2* variants sites of two GATA2 deficiency-related HLH patients in our center (Protter, http://wlab.ethz.ch/protter). Heatmap **(F)** and volcano plot **(G)** for differentially expressed genes identified comparing patient and healthy controls (HCs) (volcano plot was performed using the OmicStudio tools at https://www.omicstudio.cn). **(H)** The comparisons of plasma levels of inflammatory cytokines between the patient and HCs. **P ≤ 0.01; *P ≤ 0.05.

The physical examination revealed the presence of papules on the trunk and upper extremities. His complete blood count indicated pancytopenia with WBC (0.6-1.3)×10^9^/L, neutrophils (0.3-0.8)×10^9^/L, lymphocytes 0.2×10^9^/L, monocytes 0.00×10^9^/L, hemoglobin (72-90)g/L, and platelets (43-64)×10^9^/L. NK cells activity decreased (1.36%, normal range: ≥15.11%), and sCD25 was 6696pg/ml (normal range: <6400pg/ml). His CD107a expression in NK and CTL cells was within the normal range. His NK cell ΔPerforin was 72.56%, which was lower than the normal level. He also had elevated levels of IL-5 (76.2pg/ml, normal range: 0-17pg/ml), IFN-γ (106.3pg/ml, normal range:0-95pg/ml), ferritin (895ng/ml, normal range: 80-130ng/ml), C-reactive protein (CRP) (107mg/L, normal range: 0-8mg/L), and erythrocyte sedimentation rate (ESR) (75mm/h, normal range: 0-15mm/h). Antiphospholipid antibodies, antinuclear antibodies and antineutrophil cytoplasmic antibodies were all negative. Multiple lesions in the bilateral lung fields and multiple hypermetabolic lymphadenopathies in the hilum, mediastinum, and supraclavicular area and enlarged spleen were found by CT and PET/CT (**Figure 1A**). Mediastinum lymph node biopsy revealed a necrotic background with some plasma cells, eosinophils and lymphocytes infiltration. The pathological biopsy of subcutaneous nodules revealed lymphocyte infiltration scattered around the superficial small blood vessels of the dermis and collagen fiber proliferation. A bone marrow biopsy showed proliferation of erythroid series, predominantly of intermediate or late erythroblasts. In local hospital, the patient underwent blood cultures (including cultures for slowly-growing bacteria/mycobacteria), respiratory infection antigen IgM antibody, T-SPOT.TB, G test, GM test, Mycoplasma pneumoniae serological test, CMV-DNA and EBV-DNA, and the results were all negative. Bronchial brushing including Xpert, cryptococcal antigen, Aspergillus antigen, tuberculous smear, Fungal smear, TB-DNA, GM test were all negative. Next-generation sequencing (NGS) technology was conducted in BALF and detected *Klebsiella pneumoniae*. A diagnosis of HLH was made based on HLH-2004 criteria (8). He had no relevant family history. He had the heterozygous nonsense variant of the *GATA2* gene c.599dupG, p.S201*. His father and sister carried the wild type of the *GATA2* gene, and his mother died in an accident (**Figure 1B**).

Therapies, changes of his complete blood count, ferritin and lactate dehydrogenase during the hospitalization were shown in **Figures 1C, D**. His fever and erythema nodosa temporarily improved but relapsed after dexamethasone withdrawal. There are multiple pieces of evidence confirming the effectiveness of ruxolitinib combined with dexamethasone in the treatment of HLH (9, 10), therefore ruxolitinib was added in his treatment with dexamethasone. The blood culture at three weeks after admission revealed the presence of mycobacteria. He was finally diagnosed with HLH triggered by *Mycobacterium avium* bloodstream infection due to primary immunodeficiency caused by germline GATA2 deficiency. His symptoms did not improve despite 4-drug antituberculosis therapy, combined with dexamethasone and ruxolitinib. Bone marrow biopsy showed the hematopoietic tissue was decreased and myeloid/erythroid ratio was reduced, no typical tuberculosis granuloma or multinucleated giant cells observed. The last bone marrow smear revealed hypoplastic myelopoiesis, myeloid/erythroid ratio=0.63:1, intermediate or late erythroblasts ratio increased. Two weeks after discharge, he was hospitalized in the local hospital due to short of breath, and chest CT scan showed emerging ground glass opacities. He was given antibiotics but died of severe pulmonary infection one month later.

## Discussion

GATA2 presents two highly conserved zinc finger domains, playing a critical role during erythroid maturation and hematopoietic development. The *GATA2* variant in our patient creates stop codons that result in the absence of both two zinc finger domains (**Figure 1E**), which is the location of most pathogenic variants (6). Neither the proband’s father nor his sister carried this variant, suggesting the *GATA2* variant might be a *de novo* mutation. This mutation site has not been previously reported to be associated with GATA2 deficiency and HLH.

Current reports of HLH caused by GATA2 deficiency remain scarce, and we would venture to hypothesize that most variants of these HLH patients affected zinc finger domain function or resulted in loss of the zinc finger domain due to premature termination of translation, and the HLH onset was induced by the infections of bacteria or virus (7, 11–17) (**Table 1**). HLH occurs most commonly in infants and children, and some adults develop the disease may due to mutations with partial residual protein function that compensate for some immunological deficiencies (18). Most of the GATA2 deficiency patients presented with HLH in adulthood (11, 12, 15–17), which can be explained by partial defect in GATA2 protein function. 4 of 9 patients simultaneously experienced two zinc finger domain deletions all developed HLH after the age of 16, and were usually accompanied by rash, pancytopenia, splenomegaly and lymphadenopathy (7, 12, 16), further summary of clinical features requires the increase in the number of cases. Although it was not mentioned in other previous case reports, erythema nodosa appeared in two HLH patients with GATA2 deficiency identified as different infections in our center (7). As we know, erythema nodosa may indicate an underlying infection with non-tuberculous mycobacteria or fungi in GATA2 deficiency (4, 19), but the patient’s nodule biopsy did not reveal evidence of infection. Hence, we assumed erythema nodosa as inflammatory reaction in GATA2 deficiency patients with HLH. Furthermore, it is known that NK cells develop and function under the influence of GATA2. While GATA2 haploinsufficiency leads to a specific loss of CD56^bright^ NK cells (20), lacking activity of NK cells of this patient might be related to the absence of two zinc finger domains, with a reduced function of perforin release. The survival and differentiation potential of NK cells lacking perforin is higher (21), mutations in the perforin-coding gene cause familial HLH (22). The patient’s phenotype may suggest that the defect in the GATA2 zinc finger domain and the function of perforin secretion in NK cells needs further investigation. It was also noted that among the four nonsense mutations in the case reports, three caused loss of both zinc finger domains in GATA2 (7, 16), while one caused loss of C-terminal zinc-finger domain (17). It has been reported that 56% of GATA2 deficiency patients had lung involvement, and nontuberculous mycobacteria was the most common pathogen associated with chronic infection (23). The changes in bone marrow smear in the later stage suggest that HLH may eventually develop into MDS. Taken together, we suggest that primary immunodeficiency such as GATA2 deficiency especially patients who lack zinc finger domains should be considered in HLH patients, erythema nodosa might be an accompanying symptom, and opportunity infections should be highly suspected especially in those affecting cellular immunity (23).

**Table 1:** Summary of GATA2 deficiency patients with HLH.

Authors	Gender	Age	*GATA2* variants	Clinical Manifestations	Infection	Treatment	Prognosis
Cohen et al (11)	F	20	Unialleleic expression; heterozygous for genomic SNPs exon 2bp 55A > C and exon 3 c.15C > G, hemizygous by cDNA sequence	Fever; Lymphadenopathy; Weight loss, Dyspnea; Ulcers; Deep venous thromboses; Osteomyelitis	Enterococcus faecium bacteremia; Mycobacterium avium complex	Corticosteroids, etoposide, alemtuzumab for HLH; HSCT	Alive
Spinner et al (12)	F	18	c.871+2_3insT	Lymphedema; Fever; Headache; Pancytopenia	Herpes simplex virus-1; Blastomyces dermatitidis	Vancomycin and cefepime; vancomycin, meropenem, levofloxacin, acyclovir, and liposomal amphotericin B; dexamethasone	Death
Eguchi, et al (13)	M	14	c.1077_1078insA	Myelodysplastic syndrome; Prolonged fever; Weight loss	Mycobacterium kansasii	Unrelated BM transplantation	Remains well with full donor chimerism 16 months post-transplant.
Prader et al (14)	F	8	c.1172_1175del, p.Glu391Glyfs * 85	Abdominal pain; Rash; Fever; Pancytopenia	Varicella zoster virus	Corticosteroids, acyclovir	Relapsed after one year, recovered with corticosteroids and broad-spectrum antibiotic
Suzuki et al (15)	F	27	c.1061 C > T ,p.Thr354Met	Persistent fever; Pancytopenia; Hepatosplenomegaly; Urticarial rash	Cytomegalovirus; Methicillin-resistant staphylococcus aureus	Vancomycin; teicoplanin; daptomycin; trimethoprim/sulfamethoxazole; doxycycline; minocycline	NA
Mika et al (16)	F	29	c.177C>G,p.Tyr59*Ter	Persistent fever; Splenomegaly; Lymphadenopathy; Pancytopenia	Mycobacterium avium	alloHSCT	Complete remission
Burak et al (17)	F	22	c. 1009 C>T, p.Arg337*	Fever; Splenomegaly; Pancytopenia	Cytomegalovirus	Haploidentical human leukocyte antigen BM transplant	NA
Sun et al (7)	F	17	c.610C>T, p.Arg204*	Recurrent fever; pancytopenia; Splenomegaly; Erythema nodosa; panniculitis	Varicella zoster virus	Dexamethasone, granulocyte colony-stimulating factor	Death
Huang et al	M	28	c.599dupG, p.Ser201*	Recurrent fever, Lymphadenopathy; Pancytopenia; Splenomegaly; Erythema nodosa	Mycobacterium avium	Dexamethasone and ciclosporin; antituberculous therapy	Death

F, Female; M, Male; HLH, hemophagocyic histiocytosis; HSCT, hematopoietic stem cell transplantation; BM, bone marrow; NA, not analyzed; alloHSCT, allogeneic hematopoietic stem cell transplantation; *, termination codon.

RNA-seq was used to analyze gene expression in the proband’s peripheral blood mononuclear cells (**Figures 1F, G**), revealing that four of the top ten differentially expressed genes *SLC6A8*, *FAM210B*, *FHDC1* and *GMPR* were the targets genes of the GATA2 transcription factor in the *ChIP*-seq datasets from the ENCODE Transcription Factor Targets dataset (24, 25), which shed light on the importance of GATA2 in his phenotype. Given that the patient had an HLH phenotype, we found that it had been reported *SLC6A8* could mediate creatine to activate macrophages (26). Plasma levels of his inflammatory cytokines were significantly higher than those of the healthy controls (**Figure 1H**). Further experiments on GATA2 could not be performed because the patient was deceased.

In two cases from our center, GATA2 deficiency-associated infections were likely to be the occult cause of HLH (7). Neither patient received alloHSCT for various considerations, and respectively died at 1 and 6 months after discharge. It has been documented that alloHSCT may be a modality of a cure for GATA2 deficiency-related HLH, regardless of the presence of active infection (16). The various treatment modalities often resulted in a poor prognosis, suggesting that perhaps early alloHSCT is the treatment of choice. Therefore, having dealt with this severe disease, the only thing can be done is to save time. Early diagnosis and prompt treatment might improve the prognosis of these patients.

## Data availability statement

The datasets presented in this study can be found in online repositories. The names of the repository/repositories and accession number(s) can be found below: https://ngdc.cncb.ac.cn/gsa-human, HRA004493.

## Ethics statement

The studies involving humans were approved by the ethics committee of Peking Union Medical College Hospital. The studies were conducted in accordance with the local legislation and institutional requirements. The participants provided their written informed consent to participate in this study. Written informed consent was obtained from the participant/patient(s) for the publication of this case report.

## Author contributions

XH and BXW, writing - original draft, experiment. DW, XMH, and MS, writing - review and editing, treatment of the patient. All authors contributed to the article and approved the submitted version.
